# Association between preoperative persistent hyperglycemia and postoperative length of hospital in geriatric hip fracture patients

**DOI:** 10.1186/s12877-025-06116-z

**Published:** 2025-07-14

**Authors:** Yazhou Liu, Ying Yang, Yuhao Li, Xiaodong Yang

**Affiliations:** 1 Department of Orthopedics, Dalian Medical University, Dalian, China; 2 Department of Orthopedics, Dandong Central Hospital, Dalian Medical University, Dandong, China; 3 Department of Gynecology, Dalian Medical University, Dalian, China; 4 Department of Orthopedics, Dandong Central Hospital, China Medical University, Dandong, China

**Keywords:** Hip fracture, Length of hospital stay, Preoperative persistent hyperglycemia, Glucose levels

## Abstract

**Background:**

Metabolic disorders play a significant role in determining the length of hospital stay following hip fracture surgery in elderly patients. Therefore, it is crucial to conduct an in-depth investigation into the relationship between stress-induced hyperglycemia—one of the manifestations of metabolic disorders—and hospitalization duration in this patient population.

**Methods:**

This retrospective cohort study analyzed medical records of patients who underwent hip fracture surgery at a tertiary medical institution between January 2017 and November 2024. LASSO regression identified covariates for multivariate logistic regression. Propensity score matching minimized bias. Standardized mean differences assessed group balance. Logistic regression, restricted cubic splines, and dose-response analyses examined associations between blood glucose levels and LOS. Subgroup and threshold analyses evaluated robustness.

**Results:**

A total of 1,279 patients were included, with 673 (52.6%) having persistent preoperative hyperglycemia. The mean age was 74.7 ± 9.6 years, with 39.7% male and 60.3% female. Persistent hyperglycemia was significantly associated with prolonged LOS. Multivariate logistic regression showed a 12% increase in LOS for each unit increase in preoperative glucose (OR = 1.12, 95% CI: 1.07–1.18). Propensity score matching confirmed this association, with a significant linear trend (*P* < 0.001). Subgroup analyses revealed interactions with gender, hypertension, cerebrovascular disease, and diabetes mellitus.

**Conclusion:**

Preoperative blood glucose levels are significantly associated with prolonged LOS in elderly hip fracture patients, showing a nonlinear dose-response relationship. Each 1 mmol/L increase in glucose raises LOS risk by 12%, with stronger correlations in females and diabetics. Timely intervention is critical above 6.16 mmol/L.

**Supplementary Information:**

The online version contains supplementary material available at 10.1186/s12877-025-06116-z.

## Introduction

With the global aging population on the rise, hip fractures have emerged as a significant global challenge, becoming one of the most prevalent health issues among the elderly [1, 2]. The high incidence of hip fractures profoundly impacts mobility, independence, and overall well-being in older adults [3–5]. Current projections suggest that by 2025, the number of hip fracture cases worldwide will reach approximately 6 million. To date, early surgical intervention remains the standard treatment approach [6]. However, the post-surgical recovery and rehabilitation process is often prolonged, imposing a substantial financial burden on healthcare systems. Furthermore, studies have shown that extended hospitalization is linked to a higher early post-discharge mortality rate [7]. While current research predominantly focuses on the role of complications, malnutrition, and frailty in prolonging hospital stays, there is comparatively little attention paid to the effects of metabolic disorders on extended hospitalization following hip fracture surgery in older adults [8–10].

Hip fractures not only cause damage to the musculoskeletal system but also induce a stress response, leading to the release of stress hormones that, through neuroendocrine changes, impair insulin sensitivity. This cascade of events triggers disruptions in glucose metabolism, contributing to the development of stress-induced hyperglycemia. In many cases, hyperglycemia upon hospital admission (≥ 6.1 mmol/L) serves as an indicator of stress-induced hyperglycemia [11]and represents one of the most prevalent metabolic disorders among elderly patients with hip fractures [12, 13]. Stress-induced hyperglycemia is strongly associated with oxidative stress and inflammatory responses [14, 15]. Furthermore, sustained hyperglycemia can compromise immune function, delay wound healing, and elevate the risk of infections [16, 17]. Collectively, these factors may interact synergistically, exacerbating the length of hospital stays following hip fracture surgery in elderly patients.

Recent studies have shown that hyperglycemia, even in non-diabetic individuals, can significantly affect postoperative outcomes. For instance, Chang et al. [18]. found that stress-induced hyperglycemia was associated with prolonged hospitalization and delayed recovery in patients following major surgery. While Rizvi et al. [19]. suggested that elevated blood glucose levels contribute to longer hospital stays, their study did not isolate the role of stress-induced hyperglycemia from pre-existing diabetes. This highlights the need for further research into the impact of hyperglycemia in elderly hip fracture patients, independent of diabetes diagnosis.

The present study aims to investigate the relationship between preoperative hyperglycemia and LOS, specifically focusing on the impact of blood glucose levels rather than the diagnosis of diabetes. By filling this gap, our research seeks to provide valuable insights into preoperative blood glucose management, ultimately guiding clinical practice and improving patient outcomes. We hope that through a large-scale retrospective cohort study, we can offer clinicians accessible, timely biomarkers to help prevent extended hospital stays by effectively managing preoperative blood glucose levels.

## Methods

### Study design and data collection

This retrospective cohort study analyzed the electronic medical records of hip fracture patients at our hospital from January 2017 to November 2024. It documented baseline characteristics, as well as laboratory results from admission and within 48 h prior to surgery. Blood samples were meticulously collected, processed, and analyzed in accordance with the established protocols of an international biochemical laboratory. The data collection process was independently conducted by two authors (LYZ and YY), who diligently reviewed any discrepancies to ensure the data’s accuracy. In compliance with the ethical guidelines of the 1964 Declaration of Helsinki, the study received approval from the Institutional Review Board (IRB), and written informed consent was deemed unnecessary.

### Study population

The study participants were patients with hip fractures who underwent surgical treatment. The exclusion criteria were as follows: (1) multiple or pathological hip fractures; (2) age under 60 years; (3) absence of preoperative laboratory tests within 48 h prior to surgery or incomplete electronic medical records; (4) patients who required emergency surgery; (5) underlying conditions directly affecting hematological parameters, such as infections, cirrhosis, exogenous albumin supplementation, or leukemia; (6) incomplete admission or discharge records.

### Exposure and outcome

Persistent hyperglycemia refers to elevated blood glucose levels that meet the criteria for mild, moderate, or severe hyperglycemia in two or more consecutive tests [20]. Blood glucose levels were measured upon admission and within 48 h prior to surgery. The criteria for hyperglycemia were based on the 2023 Clinical Practice Guidelines issued by the American Diabetes Association, which classify blood glucose levels as follows: normal blood glucose < 6.1 mmol/L, mild hyperglycemia 6.1–7.8 mmol/L, moderate hyperglycemia 7.8–10.0 mmol/L, and severe hyperglycemia ≥ 10.0 mmol/L [20, 21]. In this study, persistent hyperglycemia was defined as meeting the criteria for any of these hyperglycemic categories on two or more consecutive tests, with the lowest category and its corresponding glucose value recorded for patients who fell into multiple categories.

In this study, preoperative blood glucose values of patients were categorized into four quartiles: the first quartile (Q1: 1.60–5.39 mmol/L), the second quartile (Q2: 5.40–6.10 mmol/L), the third quartile (Q3: 6.11–7.50 mmol/L), and the fourth quartile (Q4: 7.51–23.30 mmol/L).

Patients may be considered for discharge upon meeting the following criteria: (1) pain scores, such as the Visual Analog Scale (VAS), ≤ 3, indicating mild pain, and the effectiveness of oral analgesics in managing the pain; (2) the ability to independently perform bed-to-chair transfers and walk short distances (≥ 20 m) with the aid of assistive devices, such as walkers or crutches, or to complete basic daily activities with guidance from a rehabilitation therapist; (3) proper healing of wounds with no signs of infection; (4) the absence of complications, or effective management of any complications that may have occurred; (5) a strong patient request for discharge. The therapist evaluates the patient’s eligibility for discharge and collaborates with the surgeon to determine whether discharge is appropriate.

In this article, all references to length of stay denote the period from the day of surgery to the day of discharge [22]. In this study, a patient was defined as having a “prolonged hospital stay” if the length of stay exceeded the median (10 days). We observed a left-skewed distribution of LOS, indicating that the majority of patients had shorter hospital stays. Given this distribution, we used the median as the threshold for classifying a prolonged hospital stay. The median was chosen because it is a centralized trend indicator that is highly representative of skewed data.

### Covariates

In accordance with the risk factors identified in prior research, we systematically extracted relevant covariates from medical records, categorizing them into four distinct groups: demographic variables, comorbid conditions, surgery-related factors, and preoperative laboratory test results. The demographic variables encompassed age, gender, body mass index (BMI), smoking habits, and alcohol consumption. The comorbid conditions included the American Society of Anesthesiologists (ASA) classification, dementia, hypertension, diabetes mellitus, stroke, chronic obstructive pulmonary disease (COPD), cardiovascular diseases, and cerebrovascular diseases. Surgery-related factors included fracture type, surgical approach, timing of surgery, duration of surgery, blood loss, and the need for blood transfusions. Preoperative laboratory test results consisted of red blood cell count, white blood cell count, neutrophil count, lymphocyte count, hemoglobin levels, and serum albumin levels.

### Statistical analysis

We characterized patients at baseline using mean ± standard deviation or numbers (percentages). Categorical data were compared with the chi-square test, while continuous data were assessed using the Kruskal-Wallis’s test. Covariates were initially screened using a two-step approach. First, we performed univariate logistic regression analyses for all potential covariates to identify variables significantly associated with prolonged LOS. Second, we applied least absolute shrinkage and selection operator (LASSO) regression, using the binary outcome of prolonged LOS, to select predictors and reduce model overfitting. All clinically relevant variables—including demographics, comorbidities, surgical characteristics, and preoperative lab values—were entered into the LASSO model. The optimal penalty parameter (λ) was chosen via 10-fold cross-validation. Variables identified in either the univariate or LASSO analysis were entered into the final multivariate logistic regression model to calculate adjusted odds ratios (ORs) and 95% confidence intervals (CIs).To minimize confounding, propensity score matching (PSM) was performed using a 1:1 nearest-neighbor matching algorithm without replacement. A caliper width of 0.1 standard deviations of the logit of the propensity score was applied to ensure close matches. Standardized mean differences (SMD) were used to assess potential imbalances between the two groups, with an SMD ≥ 0.10 indicating a significant difference. Subsequently, logistic regression analyses were conducted on the PSM-matched data to derive adjusted ORs and 95% CIs.

Furthermore, a restricted cubic spline plot was generated to explore the relationship between continuous glucose levels and the risk of LOS. To further investigate the association between persistent preoperative hyperglycemia and LOS, regression analyses were performed to model the relationship between glucose levels and hospitalization duration, and dose-response curves were plotted. Threshold analyses were also conducted to elucidate the relationship between persistent preoperative hyperglycemia and LOS. Finally, subgroup analyses will be performed to evaluate the potential impact of other variables on the relationship between preoperative continuous glucose levels and LOS, thereby assessing the robustness of our conclusions.

All statistical tests were two-sided, with a significance level set at *P* < 0.05. Data will be analyzed using IBM SPSS Statistics (version 25.0, IBM Corp., Armonk, NY, USA) and R (version 4.3.1, R Foundation for Statistical Computing, Vienna, Austria).

## Results

### Study population characteristics

In this study, a total of 2,573 electronic medical records were collected from January 2017 to November 2024. After screening for patients who met the inclusion and exclusion criteria, 1,279 patients were included in the analysis. Among these, 673 patients (52.6%) exhibited persistent hyperglycemia preoperatively (i.e., multiple glucose readings ≥ 6.1 mmol/L), and 545 patients (42.6%) experienced prolonged length of stay. The mean age of the participants was 74.70 ± 9.55 years, with 39.72% male and 60.28% female. e-Supplementary Table 1 provides a summary of the baseline characteristics of all patients.

### Subgroup analysis based on clinical classifications

**Fig. 1:**
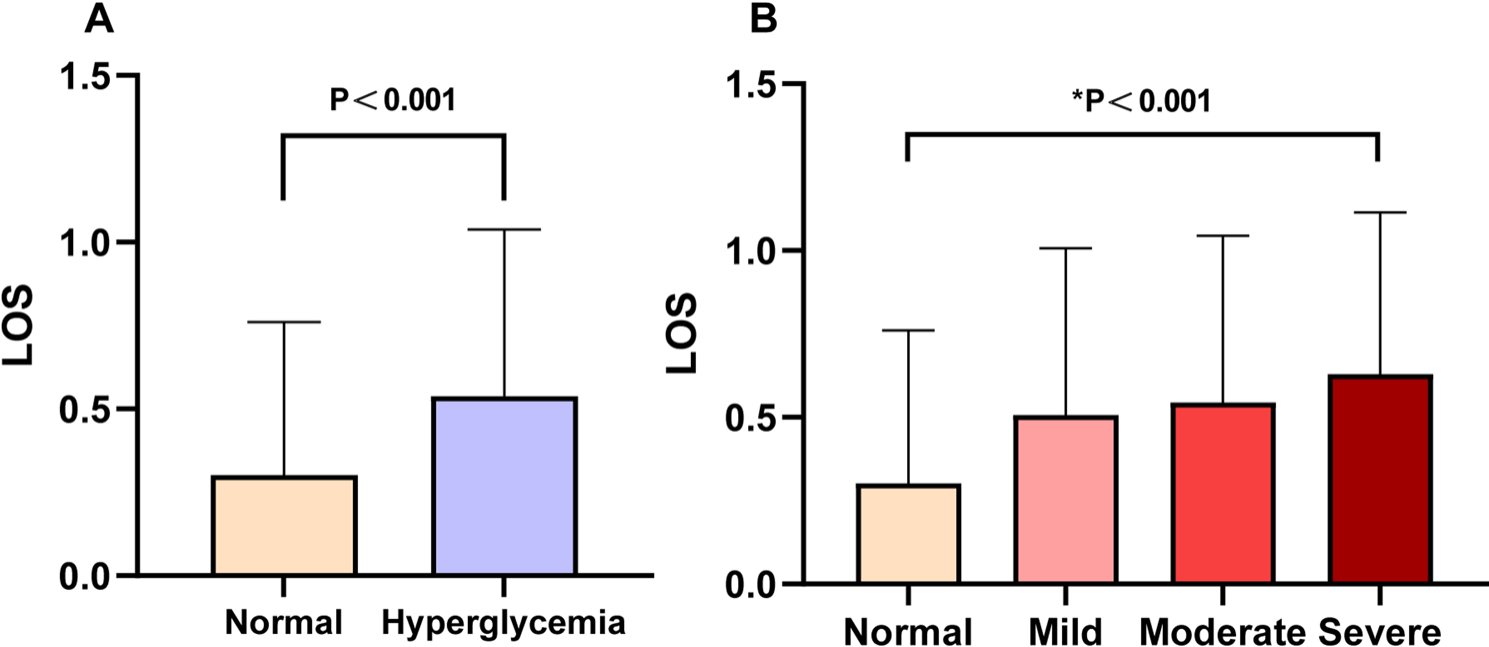
The bar graph shows the incidence of LOS associated with different persistent hyperglycemias. In panel **A**, the incidence of LOS was 30.2% in the normoglycemic group and 53.8% in the hyperglycemic group. Panel **B** shows the difference in the incidence of LOS at different clinical threshold glucose levels: 30.2% in the normoglycemic group, 50.6% in the mild hyperglycemic group, 54.1% in the moderately hyperglycemic group, and 62.9% in the severely hyperglycemic group

Subgroup analyses of persistent hyperglycemias, based on clinical practice guidelines, revealed significant differences (*p* < 0.05) across several variables, including age, gender, BMI, ASA classification, hypertension, diabetes mellitus, stroke, COPD, cardiovascular disease, cerebrovascular disease, surgical factors, and preoperative laboratory results. Furthermore, as illustrated in Fig. 1, the incidence of prolonged LOS was significantly higher in the persistent hyperglycemia group compared to the normoglycemic group (66.4% vs. 33.6%, *P* < 0.001).

### Predictive modeling and risk estimation

E-Supplementary Fig. 1 and e-Supplementary Table 2 present the odds ratios from univariate analyses, LASSO regression coefficients, and the adjusted ORs derived from the final multivariate model. Several variables with relatively weak associations in univariate analysis—such as ASA class, cerebrovascular disease, and neutrophil count (*p* < 0.05)—were retained through LASSO regression. Conversely, some clinically recognized factors were identified in the univariate analysis but were not selected by LASSO. By integrating both approaches, the final model achieved comprehensive adjustment for potential confounding factors. In the final multivariate logistic regression, persistent hyperglycemia (modeled as a continuous variable) was significantly associated with the odds of prolonged LOS. After adjustment, each 1 mmol/L increase in glucose was associated with a 12% increase in the odds of prolonged LOS (OR = 1.11; 95% CI: 1.05–1.17). We emphasize that this reflects an increase in odds, not in the actual duration of hospitalization. Specifically, each unit increase in preoperative glucose was associated with a 11% increase in the likelihood of prolonged LOS. Furthermore, irrespective of whether persistent hyperglycemias were categorized using clinical classifications or equidistant quartiles, there was a consistent trend showing a higher risk of LOS in the hyperglycemic group compared to the normoglycemic group (OR = 2.03, 95% CI: 1.58–2.61). The clinical threshold-based analysis demonstrated that patients with severe hyperglycemia had a significantly increased risk of prolonged LOS (OR = 2.70, 95% CI: 1.76–4.13). The quartile analysis further emphasized the dose-response relationship between glucose levels and LOS, with the highest quartile (Q4) showing the greatest association with prolonged hospital stay (OR = 2.61, 95% CI: 1.82–3.75). Both analyses, despite using different classification systems, yielded consistent findings, reinforcing the significant relationship between hyperglycemia and prolonged LOS.

### Propensity score matching and sensitivity analysis

To further mitigate potential confounding effects, propensity score matching (PSM) was applied in this study. After matching, 373 pairs of normoglycemic and hyperglycemic patients were successfully matched, resulting in the exclusion of 233 normoglycemic and 300 hyperglycemic patients. Covariate balance before and after matching was evaluated using SMDs, with an SMD ≥ 0.10 indicating meaningful imbalance. All covariates achieved adequate balance after matching (all SMDs < 0.1). The baseline characteristics of patients before and after matching are provided in e-Supplementary Table 3. Logistic regression analysis of the matched cohort confirmed that higher persistent hyperglycemias were significantly associated with an increased risk of LOS, demonstrating a clear linear trend (OR = 3.30, 95% CI: 1.96–5.56, *P* < 0.001 for trend, as shown in Table 1).

**Table 1:** Adjusted association between preoperative glucose levels and postoperative length of stay

Glucose (mmol/L)	Events, *n* (%)	Multivariable regression adjusted OR (95% CI)	*P*	PSM adjusted OR (95% CI)	*P*
Continuous	NA	1.11 (1.05–1.17)	< 0.001	NA	NA
Dichotomy					
Normal, < 6.1	183 (33.6%)	1 [Reference]	< 0.001	1 [Reference]	< 0.001
Hyperglycemia, ≥ 6.1	362 (66.4%)	2.03 (1.58–2.61)		1.75 (1.35–2.26)	
Clinical threshold					
Normal, < 6.1	183 (33.6%)	1 [Reference]	< 0.001*	1 [Reference]	< 0.001*
Mild, 6.1–7.8	199 (36.5%)	1.91 (1.44–2.53)		1.62 (1.22–2.17)	
Moderate, 7.8–10.0	80 (14.7%)	1.93 (1.31–2.86)		1.44 (0.94–2.19)	
Severe, ≥ 10.0	83 (15.2%)	2.70 (1.76–4.13)		3.30 (1.96–5.56)	
Quartile					
Q1(1.60–5.40)	90 (16.5%)	1 [Reference]	< 0.001*	1 [Reference]	< 0.001*
Q2(5.40–6.10)	93 (17.1%)	1.10 (0.76–1.59)		1.01 (0.73–1.41)	
Q3(6.10–7.50)	167 (30.6%)	1.81 (1.28–2.55)		2.00 (1.44–2.77)	
Q4(7.50–23.3)	195 (35.8%)	2.61 (1.82–3.75)		2.53 (1.70–3.78)	

* P for trend

NA: Not Applicable; CI: Confidence Interval; OR: Odds Ratio; PSM: Propensity Scores Matching

### Dose-Response relationship and threshold analysis

To further investigate the relationship between preoperative persistent hyperglycemia and LOS, we employed a generalized linear model (GLM) for the analysis. The results revealed that each 1-unit increase in persistent hyperglycemia significantly extended LOS by 0.54 days (95% confidence interval: 0.42–0.67, *P* < 0.001) after adjusting for potential confounding factors. In comparison to patients with normal persistent hyperglycemias, those with elevated glucose levels experienced prolonged hospitalization, with the length ranging from 4 to 7 days (mild hyperglycemia group and severe hyperglycemia group: OR = 4.01, 95% CI: 3.34–4.67, OR = 5.91, 95% CI: 4.74–7.08, refer to e-supplementary Table 4 for detailed information).

**Fig. 2:**
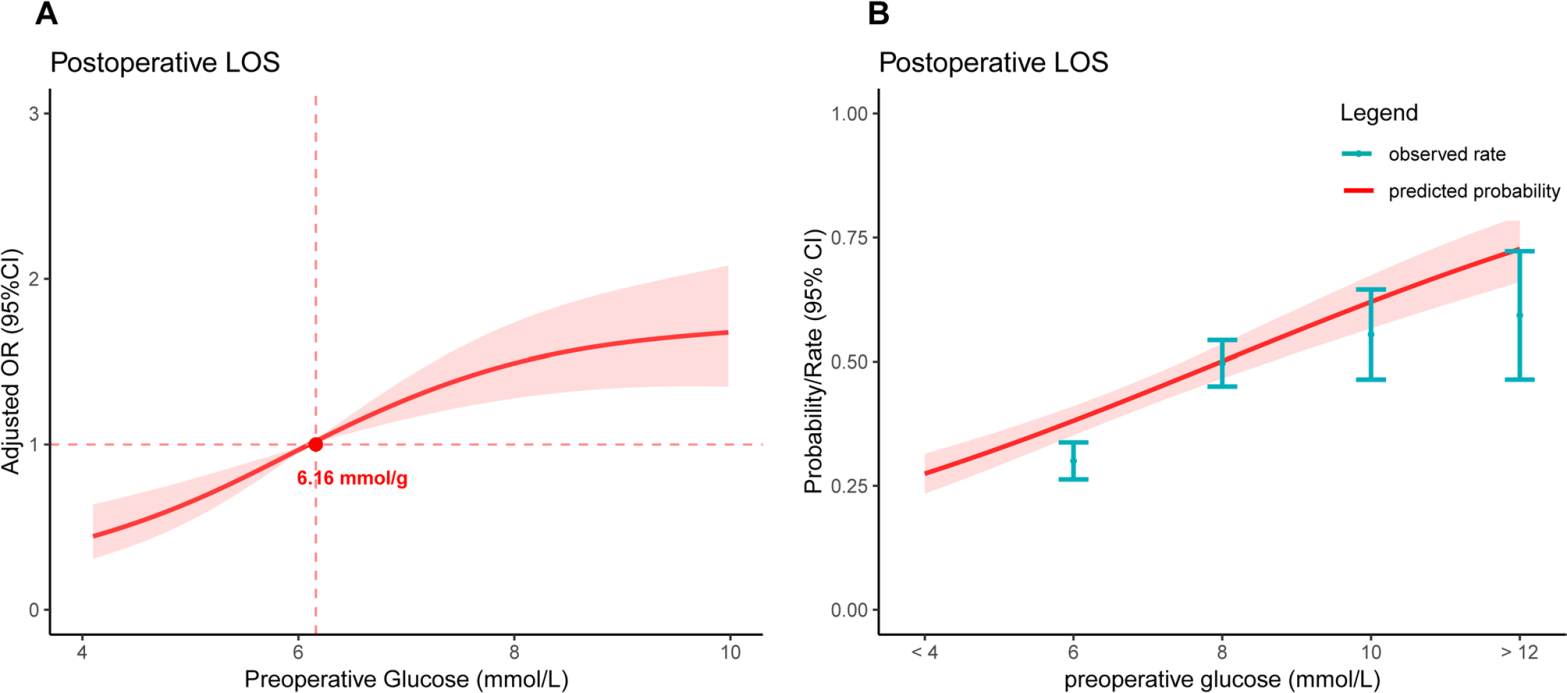
Association between persistent hyperglycemias with risk **(A)** and predicted probability **(B)** of LOS in patients with hip fractures. The restricted cubic spline plot **(A)** has been adjusted for all included covariates. The shaded areas represent the 95% confidence intervals

As depicted in Fig. 2A; after controlling for various potential confounders, the results of the restricted cubic spline regression analysis highlighted a significant positive association between preoperative persistent hyperglycemia and LOS. The OR for LOS increased markedly as preoperative continuous glucose levels rose (*P* < 0.05). To further delineate the exact nature of this relationship, we performed a threshold analysis, which confirmed a significant nonlinear association between preoperative continuous glucose and LOS (P for Log-likelihood ratio < 0.001, see Table 2 for details). Additionally, Fig. 2 illustrates the relationship between preoperative persistent hyperglycemia and the predicted probability of LOS, suggesting that higher persistent hyperglycemias are linked to a greater likelihood of prolonged hospitalization.

**Table 2:** Threshold analysis of preoperative glucose levels on postoperative length of stay in geriatric hip fracture patients

		Adjusted HR (95% CI)	*P* value	*P* for Log-likelihood ratio†
All-cause mortality				
preoperative glucose levels	Fitting by the standard linear model	1.12(1.06–1.18)	< 0.0001	
	Inflection point:6.16			< 0.0001
	Fitting by the two-piecewise linear model			
	ALI index < 6.16	1.53(1.31–1.79)	< 0.0001	
	ALI index > 6.16	1.02(0.95–1.09)	0.5917	

†Loglikelihood ratio is used to assess whether there is a statistical difference between two segmented linear models

### Interaction and stratified analyses

**Fig. 3:**
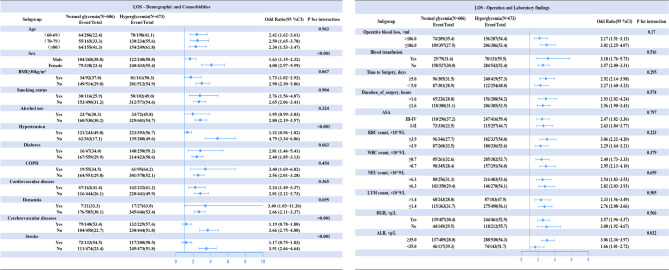
Subgroup analysis of the associations between persistent hyperglycemias and LOS, adjusted for all the covariates

Subsequently, subgroup analyses were conducted to examine the influence of other covariates on the relationship between preoperative persistent hyperglycemia and LOS (see Fig. 3). The results revealed a statistically significant interaction with gender, hypertension, cerebrovascular disease, stroke, and albumin levels (all interaction P values < 0.05). Notably, in the female population, identical preoperative persistent hyperglycemia was associated with a higher incidence of prolonged LOS in patients without hypertension, cerebrovascular disease, and a history of stroke, who also had an albumin level > 35 g/L (*P* < 0.05). Given the established link between hyperglycemia and diabetes mellitus, we further investigated the interaction between diabetes mellitus diagnosis and different hyperglycemia thresholds (for further details, refer to e-supplementary Table 5). When the hyperglycemia threshold was increased to 7.8 mmol/L, the previously nonsignificant interaction between preoperative hyperglycemia and diabetes diagnosis reversed. These findings suggest that persistent preoperative hyperglycemia (> 7.8 mmol/L) is significantly associated with an increased risk of LOS in patients with a prior diagnosis of diabetes (OR = 2.86, 95% CI: 1.76–4.65, *P* = 0.022).

## Discussion

The length of stay in patients with hip fractures presents a significant challenge in clinical management. LOS is not only a reliable predictor of post-traumatic healthcare costs but is also strongly correlated with overall healthcare expenses [23]. Research has demonstrated a direct association between prolonged LOS and an increased incidence of healthcare-associated infections [24, 25]. More critically, Nikkel et al. discovered that shorter hospital stays in hip fracture patients were significantly linked to reduced early mortality rates [26]. Furthermore, persistent elevated preoperative blood glucose levels in hip fracture patients are strongly associated with poor prognosis [27, 28]. This study further explored the relationship between persistent hyperglycemias and LOS. The findings revealed a nonlinear dose-response relationship between preoperative blood glucose levels and the duration of postoperative hospitalization in hip fracture patients. Notably, the study identified 6.16 mmol/L as a critical threshold, beyond which hyperglycemia significantly increased the risk of prolonged LOS. Threshold analysis further indicated that patients with persistent hyperglycemias below 6.16 mmol/L were significantly more likely to experience extended LOS as their glucose levels increased. Finally, the study also found that patients presenting with hyperglycemia upon admission had a 2.03-fold higher risk of prolonged LOS compared to those with normal blood glucose levels.

This study found that hip fractures are more prevalent in older women, and that persistent preoperative hyperglycemia has a more significant impact on LOS in this population. Previous literature has consistently shown that hip fractures are more common in women, particularly postmenopausal women. This is primarily attributed to the reduction in estrogen and progesterone levels, which leads to decreased BMD, thereby increasing fracture risk [29–31]. Additionally, physiological alterations associated with elevated blood glucose levels may further exacerbate adverse outcomes. In hyperglycemic conditions, elevated blood glucose impairs neutrophil function, promotes excessive production of reactive oxygen species (ROS), free fatty acids, and inflammatory mediators, and enhances the expression of pro-inflammatory cytokines. These pathophysiological changes not only cause cellular damage but also contribute to vascular and immune dysfunction, further accelerating bone resorption [32–35]. Surgical trauma intensifies the systemic inflammatory response, thereby exacerbating the interplay between these pathological mechanisms [36, 37].

In a comparison of LOS between hypertensive patients and those without hypertension, it was found that patients without hypertension exhibited a shorter LOS, a phenomenon that warrants further investigation. Previous studies have demonstrated that long-term use of antihypertensive medications can help hypertensive patients maintain a more stable level of body inflammation and oxidative stress [38]. Antihypertensive drugs not only mitigate blood pressure fluctuations but may also enhance immune system function indirectly by regulating the body’s inflammatory response. This regulation reduces the incidence of complications during hospitalization and promotes recovery [39]. Consequently, the prolonged hospitalization observed in hypertensive patients may be attributed to the pharmacological interventions employed in their treatment.

However, persistent hyperglycemia, coupled with hip fracture trauma, may surpass the body’s compensatory capacity, resulting in extended hospitalization. Acute hyperglycemia directly impairs immune cell function, wound healing, and vascular endothelial integrity via mechanisms such as advanced glycation end products (AGEs) and oxidative stress [40–42]. Hyperglycemic conditions suppress immune responses and significantly increase infection risks, thereby prolonging recovery time. Moreover, sustained hyperglycemia can lead to microangiopathy, which impairs blood supply and oxygen delivery to the wound, further hindering the healing process [43]. Collectively, these factors likely contribute to the prolonged hospitalization in hyperglycemic patients.

In patients with cerebrovascular disease and stroke, the impact of preoperative hyperglycemia may be less pronounced than in other groups. This phenomenon is likely linked to pre-existing chronic diseases and the body’s adaptive changes in patients with cerebrovascular disease and stroke. The metabolic and circulatory systems of these patients are already compromised to some degree, resulting in slower postoperative recovery. Although hyperglycemia—a common metabolic disorder—can increase infection risk and delay wound healing by impairing immune function [44, 45], the effects of preoperative hyperglycemia on recovery are attenuated in these patients due to existing hyperglycemia-related complications, such as microangiopathy and nerve damage. Thus, despite its role in postoperative recovery, the influence of preoperative hyperglycemia is relatively diminished due to the body’s chronic adaptive responses [46, 47].

In conclusion, prolonged hospitalization in hypertensive patients and those with cerebrovascular disease or stroke can be attributed to a complex interplay of factors, including long-term pharmacological interventions, alterations in physiological adaptive mechanisms due to chronic diseases, and hyperglycemia-induced immune dysfunction and impaired wound healing. These intertwined factors significantly influence recovery speed and LOS. Therefore, a comprehensive understanding of these factors is crucial for developing individualized treatment plans aimed at reducing hospital stays and optimizing the recovery process.

Acute stress-induced hyperglycemia in patients with diabetes mellitus has been shown to have a significant synergistic effect on blood glucose levels, particularly when they reach 7.8 mmol/L. This condition markedly prolongs the length of hospital stay. Bellis’s study demonstrated that patients with stress-induced hyperglycemia generally have a poorer prognosis compared to those with diabetes-related hyperglycemia, a finding that aligns with Guo’s research [48–50]. The present study confirms that the relationship between stress-induced hyperglycemia and diabetes mellitus becomes more pronounced in terms of adverse outcomes when blood glucose levels exceed 7.8 mmol/L. Therefore, the implementation of stringent glycemic control measures and prompt glucose-lowering interventions is critical for improving the prognosis of diabetic patients and reducing their adverse outcomes.

The main strength of this study lies in its innovative approach, utilizing data spanning a longer period within our healthcare institution. This study specifically explores the correlation between sustained persistent hyperglycemias and LOS in elderly patients with hip fractures. It offers new insights into the identification of risk factors associated with prolonged hospitalization in this cohort, thereby aiding healthcare professionals in recognizing high-risk groups and developing more effective preventive and therapeutic strategies. However, further research is necessary to confirm the clinical relevance and practical applicability of sustained persistent hyperglycemias in assessing the risk of prolonged hospitalization in elderly hip fracture patients.

### Limitations

This study has several limitations that should be acknowledged. First, as a retrospective observational study, inherent biases such as residual confounding, selection bias, and information bias are unavoidable. Although we employed robust statistical techniques, including propensity score matching, to adjust for observed confounders, several relevant variables were not available in our dataset, such as nutritional status, fracture severity, cognitive function, and perioperative complications. These factors may influence both glucose levels and postoperative outcomes (e.g., LOS), and their absence may contribute to residual confounding. Second, the study design did not allow for continuous monitoring of patients’ blood glucose levels during hospitalization and rehabilitation. Temporal fluctuations in glucose control, which could have influenced the risk of prolonged LOS, were not captured, thus limiting the accuracy of our findings. Third, the inclusion and exclusion criteria were designed to control for significant confounding factors but may have introduced selection bias. For example, excluding patients with infections, cirrhosis, hematological malignancies, or those undergoing emergency surgery may limit the generalizability of our findings to more complex or acute cases. Although necessary to preserve the internal validity of our study, these exclusions could bias effect estimates. Lastly, this was a single-center study, and the results may not be generalizable to other healthcare settings with different patient populations or management protocols. Further multi-center studies are needed to confirm the external validity of our findings.

## Conclusions

There is a significant association between preoperative blood glucose levels and prolonged length of stay in elderly patients with hip fractures, demonstrating a nonlinear dose-response relationship. Notably, female and diabetic patients with preoperative blood glucose levels up to 7.8 mmol/L exhibited a stronger positive correlation between blood glucose levels and LOS. Specifically, for each 1 mmol/L increase in preoperative blood glucose, the risk of prolonged LOS increases by 12%. Timely medical intervention is particularly critical when preoperative blood glucose levels exceed the optimal threshold of 6.16 mmol/L.

## Electronic supplementary material

Below is the link to the electronic supplementary material.

Supplementary Material 1

## Data Availability

The datasets used and/or analysed during the current study available from the corresponding author on reasonable request.

## Abbreviations

LOS: Length of stay
IRB: the Institutional Review Board
VAS: the Visual Analog Scale
BMI: Body mass index
ASA: the American Society of Anesthesiologists
SMD: Standardized mean differences
PSM: Propensity score matching
