# Supplementary material for: Association between preoperative persistent hyperglycemia and postoperative length of hospital in geriatric hip fracture patients
Source: BMC Geriatr. 2025 Jul 14;25:527. doi: 10.1186/s12877-025-06116-z (PMC12257712; doi:10.1186/s12877-025-06116-z)
Supplement: Supplementary file 1 — Supplementary Material 1 [file 12877_2025_6116_MOESM1_ESM.docx]

**Appendix:**

**e-Figure 1 Flow diagram of enrollment**

**e-Figure 2 Lasso Regularization Path: Coefficient Dynamics, Binomial Deviance, and Variable Selection Across Log Lambda Value**

**e-Table 1 Baseline characteristics of the patients based on preoperative glucose levels (mmol/L)**

**e-Table 2 Multivariate Analysis of Predictors for Postoperative Length of Stay**

**e-Table 3 Characteristics of patients before and after Propensity Score Matching based on** **preoperative glucose levels (normal < 6.1 mmol/L vs. low ≥ 6.1 mmol/L)**

**e-Table 4 Association Between Preoperative Blood Glucose Levels and Prolonged Postoperative Length of Stay (LOS)**

**e-Table 5 Diabetes-hyperglycemia interaction across varying cut-offs for hyperglycemia**

**e-Figure 1 Flow diagram of enrollment**

**
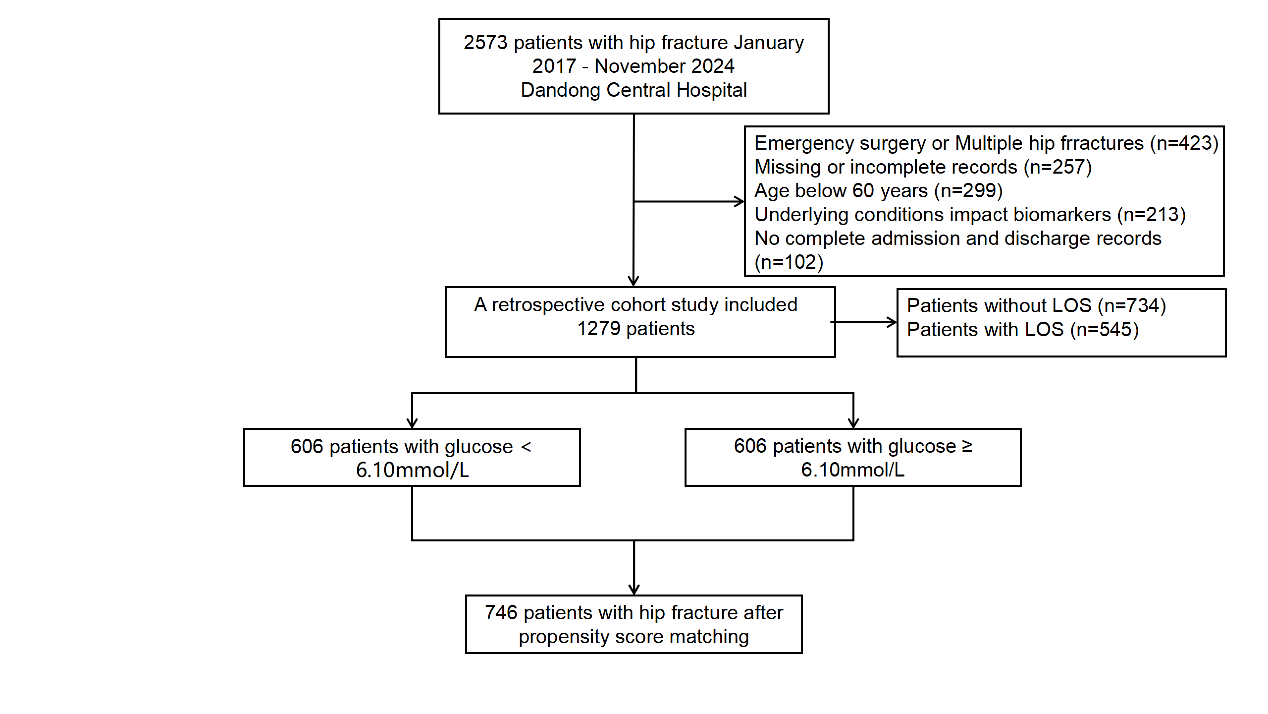
**

**e-Figure 2 Lasso Regularization Path: Coefficient Dynamics, Binomial Deviance, and Variable Selection Across Log Lambda Value**

**
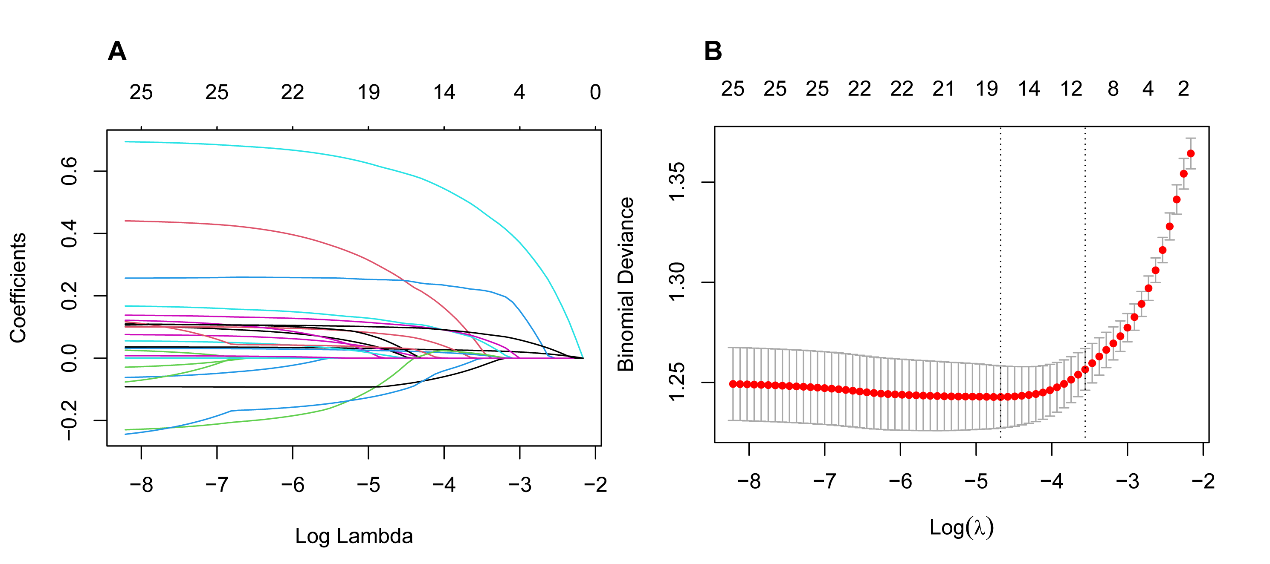
**

**e-Table1**

**Baseline characteristics of the patients based on preoperative glucose levels (mmol/L)**

| **Characteristics** | **Total patients (n = 1440)** | **Clinical Classification of Glucose levels (mmol/L)** | | | | **p** |
| --- | --- | --- | --- | --- | --- | --- |
|  |  | **Normal glucose level (<6.1, n = 606)** | **Mild Hyperglycemia (6.1–7.8, n = 393)** | **Moderate Hyperglycemia (7.8–10.0, n = 148)** | **Severe Hyperglycemia (≥ 10.0, n = 132)** |  |
| **Demographics** |  |  |  |  |  |  |
| Age, years | 74.70 ± 9.55 | 72.60 ± 9.76 | 76.34 ± 9.32 | 77.16 ± 8.94 | 76.71 ± 7.80 | <0.001 |
| Male | 508 (39.72%) | 268 (44.22%) | 154 (39.19%) | 47 (31.76%) | 39 (29.55%) | 0.002 |
| BMI ≥ 30.0 kg/m² | 253 (19.78%) | 92 (15.18%) | 83 (21.12%) | 36 (24.32%) | 42 (31.82%) | <0.001 |
| Smoking | 218 (17.04%) | 116 (19.14%) | 59 (15.01%) | 26 (17.57%) | 17 (12.88%) | 0.197 |
| Alcohol | 148 (11.57%) | 76 (12.54%) | 45 (11.45%) | 16 (10.81%) | 11 (8.33%) | 0.573 |
| **Comorbidities** |  |  |  |  |  |  |
| ASA classes ≥ III | 712 (55.67%) | 296 (48.84%) | 223 (56.74%) | 100 (67.57%) | 93 (70.45%) | <0.001 |
| Dementia | 48 (3.75%) | 21 (3.47%) | 19 (4.83%) | 8 (5.41%) | 0 (0.00%) | 0.053 |
| Hypertension | 636 (49.73%) | 243 (40.10%) | 217 (55.22%) | 94 (63.51%) | 82 (62.12%) | <0.001 |
| Diabetes | 297 (23.22%) | 47 (7.76%) | 63 (16.03%) | 83 (56.08%) | 104 (78.79%) | <0.001 |
| Stroke | 332 (25.96%) | 132 (21.78%) | 102 (25.95%) | 48 (32.43%) | 50 (37.88%) | <0.001 |
| COPD | 150 (11.73%) | 55 (9.08%) | 53 (13.49%) | 18 (12.16%) | 24 (18.18%) | 0.014 |
| Cardiovascular diseases | 394 (30.81%) | 162 (26.73%) | 120 (30.53%) | 59 (39.86%) | 53 (40.15%) | 0.001 |
| Cerebrovascular diseases | 377 (29.48%) | 148 (24.42%) | 116 (29.52%) | 58 (39.19%) | 55 (41.67%) | <0.001 |
| **Operative-related Factors** |  |  |  |  |  |  |
| Type of fracture |  |  |  |  |  | <0.001 |
| Femoral neck fracture | 684 (53.48%) | 374 (61.72%) | 184 (46.82%) | 66 (44.59%) | 60 (45.45%) |  |
| Intertrochanteric fracture | 521 (40.73%) | 200 (33.00%) | 190 (48.35%) | 70 (47.30%) | 61 (46.21%) |  |
| Subtrochanteric fracture | 74 (5.79%) | 32 (5.28%) | 19 (4.83%) | 12 (8.11%) | 11 (8.33%) |  |
| Type of surgery |  |  |  |  |  | <0.001 |
| Total Hip Arthroplasty | 162 (12.67%) | 91 (15.02%) | 34 (8.65%) | 18 (12.16%) | 19 (14.39%) |  |
| Hemiarthroplasty | 322 (25.18%) | 146 (24.09%) | 105 (26.72%) | 39 (26.35%) | 32 (24.24%) |  |
| Intramedullary nail fixation | 416 (32.53%) | 159 (26.24%) | 147 (37.40%) | 59 (39.86%) | 51 (38.64%) |  |
| Fixation with steel plate | 170 (13.29%) | 64 (10.56%) | 63 (16.03%) | 22 (14.86%) | 21 (15.91%) |  |
| Fixation with hollow nails | 209 (16.34%) | 146 (24.09%) | 44 (11.20%) | 10 (6.76%) | 9 (6.82%) |  |
| Time to surgery, days | 5.89 ± 4.02 | 5.36 ± 3.36 | 5.99 ± 4.47 | 6.09 ± 3.62 | 7.78 ± 5.13 | <0.001 |
| Duration of surgery, hours | 1.66 ± 0.80 | 1.60 ± 0.75 | 1.71 ± 0.80 | 1.66 ± 0.69 | 1.82 ± 1.09 | 0.015 |
| Operative blood loss, ml | 175.17 ± 152.54 | 162.55 ± 152.52 | 180.29 ± 135.13 | 198.62 ± 179.51 | 191.61 ± 164.45 | 0.022 |
| Blood transfusion | 210 (16.42%) | 79 (13.04%) | 76 (19.34%) | 30 (20.27%) | 25 (18.94%) | 0.021 |
| **Preoperative Laboratory Tests** |  |  |  |  |  |  |
| RBC count, ×109/L | 3.93 ± 0.68 | 3.99 ± 0.67 | 3.86 ± 0.66 | 3.83 ± 0.62 | 3.93 ± 0.79 | 0.007 |
| WBC count, ×109/L | 8.85 ± 2.87 | 8.29 ± 2.64 | 9.26 ± 2.98 | 9.15 ± 2.88 | 9.85 ± 3.07 | <0.001 |
| NEU count, ×109/L | 6.78 ± 2.79 | 6.14 ± 2.58 | 7.24 ± 2.81 | 7.24 ± 2.73 | 7.87 ± 3.06 | <0.001 |
| LYM count, ×109/L | 1.33 ± 0.67 | 1.43 ± 0.76 | 1.26 ± 0.59 | 1.24 ± 0.51 | 1.20 ± 0.52 | <0.001 |
| HGB, g/L | 119.78 ± 20.59 | 121.61 ± 20.60 | 118.53 ± 20.35 | 116.41 ± 18.40 | 118.89 ± 22.93 | 0.015 |
| Albumin, g/L | 37.95 ± 4.72 | 38.38 ± 4.63 | 37.69 ± 4.85 | 37.17 ± 4.76 | 37.62 ± 4.52 | 0.013 |

Continuous variables are presented as mean ± standard deviation, while categorical variables are represented by numbers (percentages).

BMI, Body Mass Index; COPD, chronic obstructive pulmonary disease; ASA, American Society of Anesthesiologists; RBC, Red Blood Cells; WBC, White Blood Cells; NEU, Neutrophils; LYM, Lymphocytes; HGB, Hemoglobin.

**e-Table 2**

**Multivariate Analysis of Predictors for Postoperative Length of Stay**

| **Characteristics** | **univariate** | | | **Lasso** | **Multivariate** | | |
| --- | --- | --- | --- | --- | --- | --- | --- |
|  | **OR** | **95%CI** | **p** | **Coefficient (s0)** | **OR** | **95%CI** | **p** |
| Demographics |  |  |  |  |  |  |  |
| Age, years | 1.05 | 1.04-1.07 | <0.001 | 0.0247 | 1.04 | 1.02-1.05 | <0.001 |
| Male | 1.14 | 0.91-1.42 | 0.271 | 0.0468 | 1.46 | 1.12-1.89 | 0.005 |
| BMI (≥30.0 kg/m²) | 1.16 | 0.88-1.52 | 0.307 | . | <NA> | <NA> | <NA> |
| Smoking | 0.74 | 0.55-1.00 | 0.053 | . | <NA> | <NA> | <NA> |
| Alcohol | 0.80 | 0.56-1.14 | 0.212 | . | <NA> | <NA> | <NA> |
| Comorbidities |  |  |  |  |  |  |  |
| ASA (≥III VS ＜III) | 2.03 | 1.61-2.55 | <0.001 | 0.0675 | 1.16 | 0.89-1.5 | 0.266 |
| COPD | 1.63 | 1.16-2.30 | 0.005 | . | 1.09 | 0.75-1.60 | 0.647 |
| Cardiovascular diseases | 1.85 | 1.45-2.35 | <0.001 | . | 1.12 | 0.86-1.48 | 0.403 |
| Dementia | 1.36 | 0.77-2.43 | 0.293 | . | <NA> | <NA> | <NA> |
| Hypertension | 2.59 | 2.06-3.26 | <0.001 | 0.4816 | 1.98 | 1.54-2.54 | <0.001 |
| Diabetes | 1.95 | 1.50-2.53 | <0.001 | . | 1.1 | 0.79-1.54 | 0.557 |
| Stroke | 2.19 | 1.70-2.83 | <0.001 | 0.0638 | 1.2 | 0.62-2.31 | 0.587 |
| Cerebrovascular diseases | 2.16 | 1.69-2.76 | <0.001 | 0.2155 | 1.28 | 0.68-2.40 | 0.451 |
| Operative-related Factors |  |  |  |  |  |  |  |
| Type of fracture | 0.87 | 0.73-1.05 | 0.136 | . | <NA> | <NA> | <NA> |
| Type of surgery | 0.8 | 0.73-0.88 | <0.001 | -0.0292 | 0.88 | 0.79-0.98 | 0.02 |
| Time to surgery, days | 1.06 | 1.03-1.09 | <0.001 | 0.0109 | 1.03 | 1.003-1.07 | 0.032 |
| Duration of surgery, hours | 1.07 | 0.93-1.22 | 0.365 | . | <NA> | <NA> | <NA> |
| Operative blood loss, ml | 1 | 1.00-1.001 | 0.049 | . | 1.001 | 1.00-1.001 | 0.245 |
| Blood transfusion | 1.37 | 1.02-1.84 | 0.04 | . | 0.95 | 0.66-1.38 | 0.789 |
| Preoperative Laboratory Tests |  |  |  |  |  |  |  |
| RBC count, ×109/L | 0.9 | 0.76-1.06 | 0.193 | . | <NA> | <NA> | <NA> |
| WBC count, ×109/L | 1.07 | 1.03-1.11 | 0.001 | . | 1.14 | 0.93-1.39 | 0.226 |
| NEU count, ×109/L | 1.09 | 1.04-1.13 | <0.001 | 0.0145 | 0.92 | 0.74-1.13 | 0.421 |
| LYM count, ×109/L | 0.68 | 0.56-0.83 | <0.001 | -0.0031 | 0.78 | 0.59-1.04 | 0.094 |
| HGB, g/L | 0.996 | 0.99-1.00 | 0.192 | . | <NA> | <NA> | <NA> |
| Albumin, g/L | 0.96 | 0.94-0.99 | 0.001 | . | 1.02 | 0.99-1.05 | 0.285 |
| Blood glucose, mmol/L | 1.18 | 1.13-1.24 | <0.001 | 0.0801 | 1.11 | 1.05-1.17 | <0.001 |

Coefficients are null (**.**) variables indicate that they were screened out of the Lasso regression analysis, or their coefficients were compressed to zero.

NA, Not Applicable; CI, Confidence Interval; OR Odds Ratio; BMI, Body Mass Index; ASA, American Society of Anesthesiologists; RBC, Red Blood Cells; NEU, Neutrophils; LYM, Lymphocytes; HGB, Hemoglobin.

**e-Table 3**

**Characteristics of patients before and after Propensity Score Matching based on** **preoperative glucose levels (normal < 6.1 mmol/L vs. low ≥ 6.1 mmol/L)**

| **Characteristics** | **Before matching** | | | **After matching** | | |
| --- | --- | --- | --- | --- | --- | --- |
|  | **Normal glucose (n=606)** | **Hyperglycemia (n=673)** | **SMD** | **Normal glucose (n=373)** | **Hyperglycemia (n=373)** | **SMD** |
| Demographics |  | | | | | |
| Age, years | 72.60 ± 9.76 | 76.59 ± 8.95 | 0.426 | 75.67 ± 9.61 | 75.53 ± 9.44 | 0.015 |
| Male | 268 (44.22%) | 240 (35.66%) | 0.175 | 153 (41.02%) | 157 (42.09%) | 0.022 |
| BMI ≥30.0 kg/m² | 92 (15.18%) | 161 (23.92%) | 0.222 | 64 (17.16%) | 58 (15.55%) | 0.043 |
| Smoking | 116 (19.14%) | 102 (15.16%) | 0.106 | 63 (16.89%) | 67 (17.96%) | 0.028 |
| Alcohol | 76 (12.54%) | 72 (10.70%) | 0.057 | 42 (11.26%) | 48 (12.87%) | 0.049 |
| Comorbidities |  | | | | | |
| ASA classes ≥ III | 296 (48.84%) | 416 (61.81%) | 0.263 | 213 (57.10%) | 216 (57.91%) | 0.016 |
| Dementia | 21 (3.47%) | 27 (4.01%) | 0.029 | 18 (4.83%) | 17 (4.56%) | 0.013 |
| Hypertension | 243 (40.10%) | 393 (58.40%) | 0.372 | 188 (50.40%) | 186 (49.87%) | 0.011 |
| Diabetes | 47 (7.76%) | 250 (37.15%) | 0.752 | 46 (12.33%) | 48 (12.87%) | 0.016 |
| Stroke | 132 (21.78%) | 200 (29.72%) | 0.182 | 101 (27.08%) | 100 (26.81%) | 0.006 |
| COPD | 55 (9.08%) | 95 (14.12%) | 0.158 | 43 (11.53%) | 41 (10.99%) | 0.017 |
| Cardiovascular diseases | 162 (26.73%) | 232 (34.47%) | 0.168 | 117 (31.37%) | 119 (31.90%) | 0.012 |
| Cerebrovascular diseases | 148 (24.42%) | 229 (34.03%) | 0.212 | 114 (30.56%) | 111 (29.76%) | 0.018 |
| Operative-related Factors |  | | | | | |
| Type of fracture |  |  | 0.277 |  |  | 0.004 |
| Femoral neck fracture | 374 (61.72%) | 310 (46.06%) |  | 200 (53.62%) | 196 (52.55%) |  |
| Intertrochanteric fracture | 200 (33.00%) | 321 (47.70%) |  | 148 (39.68%) | 155 (41.55%) |  |
| Subtrochanteric fracture | 32 (5.28%) | 42 (6.24%) |  | 25 (6.70%) | 22 (5.90%) |  |
| Type of surgery |  |  | 0.139 |  |  | 0.033 |
| Total Hip Arthroplasty | 91 (15.02%) | 71 (10.55%) |  | 47 (12.60%) | 41 (10.99%) |  |
| Hemiarthroplasty | 146 (24.09%) | 176 (26.15%) |  | 106 (28.42%) | 107 (28.69%) |  |
| Intramedullary nail fixation | 159 (26.24%) | 257 (38.19%) |  | 121 (32.44%) | 116 (31.10%) |  |
| Fixation with steel plate | 64 (10.56%) | 106 (15.75%) |  | 44 (11.80%) | 60 (16.09%) |  |
| Fixation with hollow nails | 146 (24.09%) | 63 (9.36%) |  | 55 (14.75%) | 49 (13.14%) |  |
| Time to surgery, days | 5.36 ± 3.36 | 6.36 ± 4.49 | 0.251 | 5.83 ± 3.56 | 5.92 ± 4.60 | 0.024 |
| Duration of surgery, hours | 1.60 ± 0.75 | 1.72 ± 0.84 | 0.154 | 1.69 ± 0.82 | 1.69 ± 0.90 | 0.007 |
| Operative blood loss, ml | 162.55 ± 152.52 | 186.54 ± 151.77 | 0.158 | 176.21 ± 165.48 | 178.10 ± 144.99 | 0.012 |
| Blood transfusion | 79 (13.04%) | 131 (19.47%) | 0.175 | 60 (16.09%) | 59 (15.82%) | 0.007 |
| Preoperative Laboratory Tests |  | | | | | |
| RBC count, ×109/L | 3.99 ± 0.67 | 3.87 ± 0.68 | 0.183 | 3.88 ± 0.64 | 3.88 ± 0.64 | 0.020 |
| WBC count, ×109/L | 8.29 ± 2.64 | 9.35 ± 2.98 | 0.377 | 8.76 ± 2.59 | 8.78 ± 2.45 | 0.009 |
| NEU count, ×109/L | 6.14 ± 2.58 | 7.36 ± 2.85 | 0.448 | 6.74 ± 2.48 | 6.78 ± 2.27 | 0.019 |
| LYM count, ×109/L | 1.43 ± 0.76 | 1.24 ± 0.56 | 0.280 | 1.28 ± 0.56 | 1.29 ± 0.60 | 0.017 |
| HGB, g/L | 121.61 ± 20.60 | 118.14 ± 20.47 | 0.169 | 118.52 ± 20.24 | 118.91 ± 19.52 | 0.020 |
| Albumin, g/L | 38.38 ± 4.63 | 37.56 ± 4.76 | 0.173 | 37.56 ± 4.40 | 37.51 ± 4.76 | 0.010 |

Continuous variables are presented as mean ± standard deviation, while categorical variables are represented by numbers (percentages).

SMD, Standardized Mean Difference; CI, Confidence Interval; OR Odds Ratio; BMI, Body Mass Index; COPD, chronic obstructive pulmonary disease; ASA, American Society of Anesthesiologists; RBC, Red Blood Cells; WBC, White Blood Cells; NEU, Neutrophils; LYM, Lymphocytes; HGB, Hemoglobin.

**e-Table 4**

**Association Between Preoperative Blood Glucose Levels and Prolonged Postoperative Length of Stay (LOS)**

| **BG** | |  | **Model1❊** |  |  | **Model2❊** |  |
| --- | --- | --- | --- | --- | --- | --- | --- |
|  |  |  | **β (95% CI)** | **p value** |  | **β (95% CI)** | **p value** |
| Continuous data |  |  | 0.54 (0.42–0.67) | <0.0001 |  | 0.89(0.76–1.03) | <0.0001 |
| Dichotomous data |  |  | 4.66 (4.07–5.26) |  |  | 4.92 (4.33–5.52) |  |
| Clinical threshold | Normal, <6.1 |  | Reference |  |  | Reference |  |
|  | Mild, 6.1–7.8 |  | 4.20 (3.56–4.84) | <0.0001 |  | 4.01 (3.34–4.67) | <0.0001 |
|  | Moderate, 7.8–10.0 |  | 6.32 (5.36–7.28) | <0.0001 |  | 6.96 (5.36–7.28) | <0.0001 |
|  | Severe, ≥ 10.0 |  | 5.09 (4.00–6.17) | <0.0001 |  | 5.91 (4.74–7.08) | <0.0001 |

**CI, Confidence Interval.**

**❊Model 1, Multivariate Generalized Linear Modeling. Model 2, Multivariate generalized linear modeling after propensity score matching.**

**e-Table 5**

**Diabetes-hyperglycemia interaction across varying cut-offs for hyperglycemia**

| **Cut-off for hyperglycemia** | **6.1 mmol/L**  **OR (95% CI)** | **p for interaction** | **7.8 mmol/L**  **OR (95% CI)** | **p for interaction** |
| --- | --- | --- | --- | --- |
| Patients with diabetes | 2.81 (1.46−5.41) | 0.663 | 2.86(1.76-4.65) | 0.022 |
| Patients without diabetes | 2.40 (1.85−3.13) |  | 1.34(0.87-2.05) |  |

CI: Confidence Interval; OR: Odds Ratio
